# Mesenchymal Stem Cells Early Response to Low-Dose Ionizing Radiation

**DOI:** 10.3389/fcell.2020.584497

**Published:** 2020-12-14

**Authors:** Marina Konkova, Margarita Abramova, Andrey Kalianov, Elizaveta Ershova, Olga Dolgikh, Pavel Umriukhin, Vera Izhevskaya, Sergey Kutsev, Natalia Veiko, Svetlana Kostyuk

**Affiliations:** ^1^Department of Molecular Biology, Research Centre for Medical Genetics, Moscow, Russia; ^2^I.M. Sechenov First Moscow State Medical University, Department of Normal Physiology, Moscow, Russia; ^3^P.K. Anokhin Institute of Normal Physiology, Moscow, Russia

**Keywords:** LDIR, ROS, MSC, adaptation, gene expression

## Abstract

**Introduction:**

Mesenchymal stem cells (MSCs) are applied as the therapeutic agents, e.g., in the tumor radiation therapy.

**Purpose of the Study:**

To evaluate the human adipose MSC early response to low-dose ionizing radiation (LDIR).

**Materials and Methods:**

We investigated different LDIR (3, 10, and 50 cGy) effects on reactive oxygen species production, DNA oxidation (marker 8-oxodG), and DNA breaks (marker ɣ H2AX) in the two lines of human adipose MSC. Using reverse transcriptase–polymerase chain reaction, fluorescence-activated cell sorting, and fluorescence microscopy, we determined expression of genes involved in the oxidative stress development (*NOX4*), antioxidative response (*NRF2*), antiapoptotic and proapoptotic response (*BCL2*, *BCL2A1*, *BCL2L1*, *BIRC2*, *BIRC3*, and *BAX1*), in the development of the nuclear DNA damage response (DDR) (*BRCA1*, *BRCA2*, *ATM*, and *P53*). Cell cycle changes were investigated by genes transcription changes (*CCND1*, *CDKN2A*, and *CDKN1A*) and using proliferation markers KI-67 and proliferating cell nuclear antigen (PCNA).

**Results:**

Fifteen to 120 min after exposure to LDIR in MSCs, transient oxidative stress and apoptosis of the most damaged cells against the background of the cell cycle arrest were induced. Simultaneously, DDR and an antiapoptotic response were found in other cells of the population. The 10-cGy dose causes the strongest and fastest DDR following cell nuclei DNA damage. The 3-cGy dose induces a less noticeable and prolonged response. The maximal low range dose, 50 cGy, causes a damaging effect on the MSCs.

**Conclusion:**

Transient oxidative stress and the death of a small fraction of the damaged cells are essential components of the MSC population response to LDIR along with the development of DDR and antiapoptotic response. A scheme describing the early MSC response to LDIR is proposed.

## Introduction

The study of the low-dose ionizing radiation (LDIR) biological effects is the focus of scientific research in radiobiology because of the variable unavoidable impacts on the human cells. People commonly exposed to LDIR over natural background levels may be exposed for medical diagnostics or accidentally. During the early decades of the 20th century, a consensus was achieved that the most fundamental radiation responses have a threshold (Vaiserman, 2010). However, subsequently, the dose–response model was replaced with a conservative model with the linear no-threshold (LNT) hypothesis that there is no threshold of radiation response (ICRP, 2007; Seong et al., 2016), and, accordingly, even the smallest ionizing radiation (IR) doses may potentially increase the cancer risk. The study of the LDIR effects revealed phenomena that do not fit into the traditional concepts of direct radiation DNA damage. There are a growing number of experimental and epidemiological evidences, which extend beyond the LNT model for cancer risks assessment at low doses (Calabrese and O’Connor, 2014).

A number of studies have shown the LDIR effect on gene expression that controls apoptosis, cell cycle progression, proliferation, and differentiation (Bajinskis et al., 2011; Liang et al., 2011). In addition, when low doses were used, a disproportionate response to different doses was found (Liang et al., 2011). A number of epidemiological studies were conducted for LDIR exposures below 10 cGy on stochastic events such as cancer incidence and effects on heredity (Tao et al., 2000; Jaikrishan et al., 2013), and it was reported that 6 cGy of LDIR exposure might increase threefold the risk of brain cancer (Pearce et al., 2012).

The response of human and mouse stem cells to large and small radiation doses is being actively studied. Most attention is drawn to embryonic stem cells, which have the great therapeutic potential. However, embryonic stem cells also have the highest sensitivity to radiation. Human embryonic stem cells respond to DNA damage by the rapid induction of caspases, phospho-H2AX foci, phosphorylation of p53, and cell cycle arrest in G2 (Qin et al., 2007; Filion et al., 2009).

Adult stem cells tend to be more resistant to cell death following damage than embryonic stem cells, although the exact mechanisms are not fully understood (Liu et al., 2014; Jacobs et al., 2016). Intrinsic differences in stem cells of different origins might determine, at least in part, their response to IR (Insinga et al., 2013; Licursi et al., 2020). Mesenchymal stem cells (MSCs) are resistant to high doses of radiation (Chen et al., 2006; Fekete et al., 2015; Nicolay et al., 2015; He et al., 2019) and retain their defining stem cell characteristics after exposure to IR (Nicolay et al., 2013). MSCs derived from adipose tissue possess a significantly stronger radiation resistance capacity than MSCs derived from umbilical cord and gingival (He et al., 2019). After IR exposure, MSCs derived from adipose tissue showed a robust and time-efficient radiation-induced DDR, stable phenotypical characteristics, and multilineage differentiation potential (Maria et al., 2016; Cho et al., 2017). On the other hand, it was shown that MSCs from adipose tissue, bone marrow, and umbilical cord exhibited a relative radioresistance independent of their tissue of origin (Rühle et al., 2018).

DDR represents the main network of signaling pathways that enable cells to respond to IR (Sugrue et al., 2013; Nicolay et al., 2015). Several studies analyzed the activity of DNA double-strand break repair in MSCs and found efficient repair of these lesions after irradiation as measured by phosphorylated histone H2AX (γH2AX) levels (Prendergast et al., 2011; Oliver et al., 2013). On the other hand, it was shown that MSCs are sensitive to very low levels of radiation and trigger senescence due to impaired autophagy and DNA repair capacity (Alessio et al., 2015). Other authors confirm that the small radiation doses induce unrepaired double-strand breaks in MSCs (Osipov et al., 2015; Pustovalova et al., 2017; Ulyanenko et al., 2019). The authors of one of the articles conducted a comprehensive analysis of time- and dose-dependent patterns of gene expression in a human MSC line exposed to IR (Jin et al., 2008). They found transcription of the different genes associated with different signaling pathways in MSCs depending on the radiation dose (0.01–1 Gy) and the cultivation duration after an irradiation (1–48 h).

Analysis of the literature has shown that the main focus is on the study of the high doses’ effects of radiation on MSCs, because it is of great practical importance for the use of stem cells in cancer therapy. The authors mainly analyze the long-term effects of radiation on MSCs (more than a few hours after the exposure). The MSC response to LDIR, especially an early response, is less studied. It is not clear how long reactive oxygen species (ROS) are produced after LDIR and what processes in MSCs maintain ROS levels after irradiation when superactive short-lived DNA-damaging radicals are already inactivated. However, a number of data indicate that oxidative stress is a necessary condition for an adaptive response and a bystander effect development in irradiated cells (Ermakov et al., 2009; Murley et al., 2018). The adaptive response increased the resistance of exposed cells to high doses of radiation (Tang and Loke, 2015; Cuttler, 2020). The bystander effect involves the transfer of information from irradiated to nonirradiated cells (Tang and Loke, 2015; Verma and Tiku, 2017; Yahyapour et al., 2018). LDIR is known to induce both an adaptive response and a bystander response in cell populations (Ermakov et al., 2009; Tang and Loke, 2015).

In our study, we investigated the early response of MSCs (15 min to 2 h after irradiation) to LDIR (3, 10, and 50 cGy). We have shown that the early MSC response involves short-term oxidative stress due to activation of pro-oxidative systems (increased *NOX4* expression) and blocking of anti-oxidative systems [transcription factor nuclear factor erythroid 2–related factor 2 (NRF2)]. Oxidative stress is accompanied by the death of the most damaged cells.

A scheme describing the response of the MSC population to LDIR is proposed as a result of this and our previous studies.

## Materials and Methods

### Cell Culture

Mesenchymal stem cells (#2303 and #2278) were retrieved from the biospecimen collection maintained by the Research Centre for Medical Genetics. MSCs were obtained from adipose tissue of patients subjected to regular surgery (Loseva et al., 2012). The MSC cultures had characteristic fibroblast-like morphology and were validated by the surface protein expression analysis (CD34^–^, CD45^–^, HLA-ABC^+^, HLA-DR^–^, CD44^+^, CD29^+^, CD49b low, CD54 low, CD90^+^, CD106^–^, CD105 low, and CD117^–^). MSCs were cultivated at 37°C in AmnioMax C-100 basal medium (Gibco), containing AmnioMax Supplement C-100. Before treatments, cells were split no more than four times.

### Irradiation of the Cells

Cells were irradiated at 20°C using pulsed roentgen radiation unit ARINA-2 (Spectroflash, Russia). The amplitude of voltage on the X-tube was 160 kV, peak energy in the radiation spectrum was 60 kV, and dose rate amounted to 0.16 Gy/min.

### MTT Test

Mesenchymal stem cells were cultured in a 96-well plate and were irradiated (different doses were applied on separate plates). The cells were cultured for 3 days after irradiation. Survival was measured using the 3-(4,5-dimethylthiazol- 2-yl)-2,5-diphenyltetrazolium bromide (MTT) assay, as described previously (Vistica et al., 1991). The plates were read at 570 nm on multimode plate reader “EnSpire” (PerkinElmer, Finland).

### Fluorescence-Activated Cell Sorting

Cells were washed in Versene solution and then treated with 0.25% trypsin under control of light microscopy. Cells were transferred to the Eppendorf tube and washed with Dulbecco modified eagle medium and then centrifuged and resuspended in phosphate-buffered saline (PBS). Cells were fixed in 3% paraformaldehyde (PFA) for 10 min at 37C, washed with PBS, and then permeabilized with 0.1% Triton X-100 (Sigma) in PBS for 15 min at room temperature. Cells were analyzed using CyFlow Space (Partec, Germany); each experiment was repeated at least three times. Subpopulations of the cells were gated as recommended by the CyFlow software.

The following antibodies were used: *γ*H2AX- Dylight488 (pSer139) (NB100-78356G, Novus Biologicals); 8OHDG (Sc-66036, Santa Cruz Biotechnology); NOX4 (Sc-30141, Santa Cruz Biotechnology); NRF2 (ab137550); pNRF2 (Ser40) (Bioss Inc; BS-2013R), BRCA2 (NBP1-88361, Novus Biologicals); proliferating cell nuclear antigen (PCNA) (ab2426, Abcam); ATM; P53; PPARG; KI-67 fluorescein isothiocyanate (FITC) (sc-23900 FITC) (Santa Cruz Biotechnology); and BCL2 (Sc-783, Santa Cruz Biotechnology).

Using fluorescence-activated cell sorting (FACS), we encountered a problem that complicates the irradiated cell analysis. It turned out that MSCs (50 cGy) were more sensitive to the procedures of the cell preparation for analysis than MSCs control (C), MSCs 3 cGy, or MSCs 10 cGy. During the routine isolation and fixation procedure, including cell washing, treatment with trypsin and EDTA solutions, and centrifugation, damaged cells were lost (detached from the carrier, partially destroyed and less precipitated by centrifugation), which resulted in the least damaged cells selection. In a number of experiments, we obtained the paradoxical results – MSC damage by 50 cGy was lower than by 3 cGy. Therefore, the sample preparation protocol has been changed. We additionally isolated the cells from all washing solutions by the centrifugation. The cells of the population MSC (50 cGy) required a longer precipitation from solutions. The completeness of cells sedimentation during centrifugation was monitored. In addition, when preparing the samples for fixed MSC (50 cGy), we reduced the concentration of Triton X-100 to 0.05%.

### Annexin V Binding Assays

Cells were detached, washed with PBS, and treated with annexin V–FITC I in buffer (10 mM HEPES, pH 7.4, 140 mM NaCl, 2.5 mM CaCl_2_) at 20°C for 15 min and immediately analyzed using an automated cell counter (Countess II FL, Thermo Fisher) or FACS (CyFlow Space).

### ROS Assays

#### FACS ROS Assay

Irradiated or control cells were washed with PBS and incubated with 10 μM H2DCFH-DA (Invitrogen) at 37°C in the dark for 20 min. Cells were detached, washed with PBS, and immediately analyzed by FACS.

#### Fluorescence Microscopy

Cells were grown in slide flasks. Irradiated or control cells were washed with PBS and incubated with 10 μM H2DCFH-DA at 37°C in the dark for 20 min. Cells were washed 2× with PBS and immediately photographed.

#### Plate ROS Assay

Cells were grown in 96-well plates (Nunclon, Germany). Irradiated or control cells were incubated with 10 μM H2DCFH-DA at 37°C in the dark for 20 min. Fluorescence was measured with λex = 490 nm and λem = 524 nm (EnSpire, PerkinElmer, Finland).

### Fluorescence Microscopy

Fluorescence microscopy was carried out as described previously (available online at^1^,^2^). The following antibodies were used: *γ*H2AX (pSer139), NOX4 (Sc-30141), and NRF2 (ab137550); BCL2 (Sc-783). Rhodamine phalloidin (Molecular Probes/Invitrogen, CA, United States) was used to label cytoskeletal F-actin according the method described (Wieland et al., 1983). Images were obtained using an AxioScope A1 microscope (Carl Zeiss).

### Quantification of mRNA Levels

Quantification of mRNA levels was carried out as described previously (available online at see text footnote 1). The following primers were used:

NOX4 (F: TTGGGGCTAGGATTGTGTCTA, R: GAGTGTT CGGCACATGGGTA), BCL2 (F: TTTGGAAATCCGACCACT AA, R: AAAGAAATGCAAGTGAATGA), BCL2A1 (F: TACA GGCTGGCTCAGGACTAT, R: CGCAACATTTTGTAGCACT CTG), BCL2L1 (F: CGACGAGTTTGAACTGCGGTA, R: GGG ATGTCAGGTCACTGAATG), CCND1 (F: TTCGTGGCCTCT AAGATGAAGG, R: GAGCAGCTCCATTTGCAGC), CDKN2A (F: ATGGAGCCTTCGGCTGACT, R: GTAACTATTCGGTG CGTTGGG), BRCA1 (F: GGCTATCCTCTCAGAGTGACATT TTA, R: GCTTTATCAGGTTATGTTGCATGGT), BIRC2 (F: GA ATCTGGTTTCAGCTAGTCTGG, R: GGTGGGAGATAATGA ATGTGCAA), BIRC3 (F: AAGCTACCTCTCAGCCTACTTT, R: CCACTGTTTTCTGTACCCGGA), BAX (F: CCCGAGA GGTCTTTTTCCGAG, R: CCAGCCCATGATGGTTCTGAT), BRCA2 (F: CCTCTGCCCTTATCATCACTTT, R: CCAGAT GATGTCTTCTCCATCC), and TBP (reference gene) (F: GCC CGAAACGCCGAATAT, R: CCGTGGTTCGTGGCTCTCT).

### Comet Assay

A cell suspension in low-melting-point agarose was dropped onto slides precoated with 1% normal-melting-point agarose. The slides were placed in a solution (10 mM Tris-HCl, pH 10, 2.5 M NaCl, 100 mM EDTA, 1% Triton X-100, 10% dimethyl sulfoxide, 4°C, 1 h) and then in electrophoresis buffer (300 mM NaOH, 1 mM EDTA, pH > 13). Electrophoresis was performed for 20 min at 1 V/cm, 300 mA. The slides were fixed in 70% ethanol and stained with propidium iodide (PI) (Sigma) (2 μg/mL in PBS).

### Statistics

All the reported results were reproduced at least three times as independent biological replicates. In FACS, the median of signal intensities was analyzed. The figures show the mean and standard deviation (SD) values. The significance of the observed differences was analyzed with nonparametric Mann–Whitney *U* tests. *P* < 0.05 was considered statistically significant and is marked in the figures with ^∗^. Data were analyzed with StatPlus 2007 professional software^3^.

### Ethics

The study design was reviewed and approved by the Local Ethics Committee of FSBI “RSMG” (Federal State Budgetary Institution “Research Centre for Medical Genetics”) to meet the requirements of the Helsinki Declaration of 1975 as revised in 2013. An informed consent for the use of the surgical material had been obtained from each patient, from whom an anonymous cell culture was derived.

## Results

This study was performed using MSC lines (#2278 and #2303) obtained from adipose tissue of two different donors and characterized by their CD marker expression (Loseva et al., 2012). Subconfluent cells (approximately 70–80% confluence) were irradiated with ionizing x-ray radiation at doses of 3, 10, or 50 cGy. Populations of control and irradiated cells were designated MSCs (C), MSCs (3 cGy), MSCs (10 cGy), and MSCs (50 cGy), respectively.

Low-dose ionizing radiation appeared to exert both a direct effect on cellular structures via hitting with an energy quantum or particle and an indirect effect mediated by free radicals. The process of ionization was accompanied by the synthesis of active and short-living free radicals. These radicals quickly damaged the cellular DNA (Collinson et al., 1960; Cadet et al., 2010).

Thus, to assess the MSC response to LDIR, we first analyzed the ROS level and the cellular DNA damage. Analyzing the ROS level in unfixed cells, we found an unusual MSC property complicating irradiated cell analysis by fluorescence methods: the autofluorescence significantly increased in irradiated unfixed MSCs. We have investigated this MSC response to LDIR action in details.

### LDIR Induced MSC Autofluorescence

**FIGURE 1:**
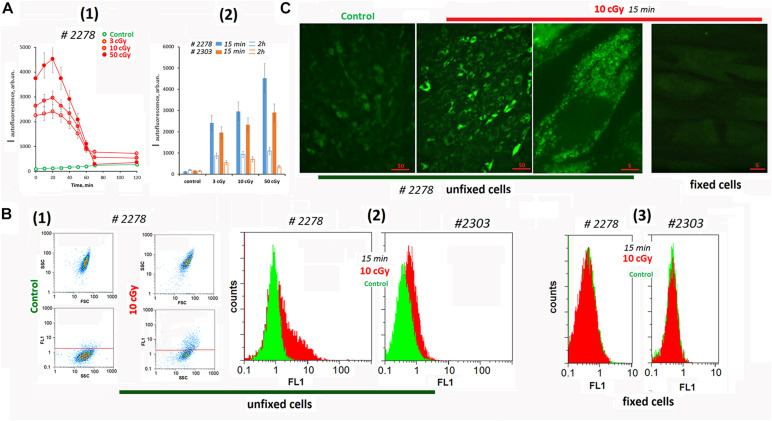
Autofluorescence of the irradiated cells. **(A)** Plate assay (λex = 490 nm and λem = 524 nm). (1) Time dependence of the signal after irradiation with doses of 3, 10, and 50 cGy. (2) Comparison of two cell lines by signal intensity 15 min and 2 h after irradiation. Average values are given for two experiments. In each experiment; cells were analyzed in four wells of the plate. **(B)** FACS. (1) Plots: SSC versus FSC and FL1 versus SSC. (2) The distribution of the unfixed MSCs with varying autofluorescence. (3) The distribution of the fixed MSC (10 cGy) with varying autofluorescence. The experiment was repeated three times. **(C)** Fluorescence microscopy of irradiated unfixed and fixed with 3% paraformaldehyde MSCs. The experiment was repeated three times.

Analyzing cells #2278 and #2303 fluorescence using a plate reader, we found a change in the background signal in the irradiated cells (Figure 1A). Autofluorescence increased significantly after irradiation and then decreased.

To determine the signal source (the culture medium or the cells themselves), we analyzed the irradiated cells using the FACS method (Figure 1B). Irradiation significantly increased the fluorescence (FL1 and FL2 channels) of the entire cell population. At the same time, we observed a subpopulation of cells #2278 (∼30%) with an abnormally high fluorescence level (Figure 1B2). In formaldehyde-fixed cells, the signals of control cells did not differ from those of irradiated cells (Figure 1B3).

Fluorescence microscopy confirms a high autofluorescence in irradiated unfixed MSCs (Figure 1C and Supplementary Figure 1A). Autofluorescence was observed in a wide range of wavelengths and exclusively in the cytoplasm of the cells. In fixed cells, autofluorescence was completely absent.

To understand whether the observed response of unfixed MSCs is specific to the LDIR effect, we conducted additional experiments (Supplementary Figure 1B). The cells were irradiated with UV with a wavelength longer than 320 nm (UVa). UVa did not directly damage the DNA of cells, but induced the ROS synthesis. For comparison, cultured adult skin fibroblasts (HSFs) were used in the experiment. In UVa-irradiated MSCs, autofluorescence increased 10-fold compared to the control. Autofluorescence was not observed in fixed UVa-irradiated MSCs. In UVa-irradiated HSFs, we did not observe an increase in autofluorescence.

Thus, we found a specific response of MSCs to LDIR or UV irradiation, which induced ROS synthesis. Unlike other fibroblast-like cells (HSFs), in MSC irradiation stimulated a significant increase of autofluorescence. This response should be taken into account for unfixed irradiated MSCs analysis by fluorescence methods.

### LDIR Induced ROS Production in MSCs

**FIGURE 2:**
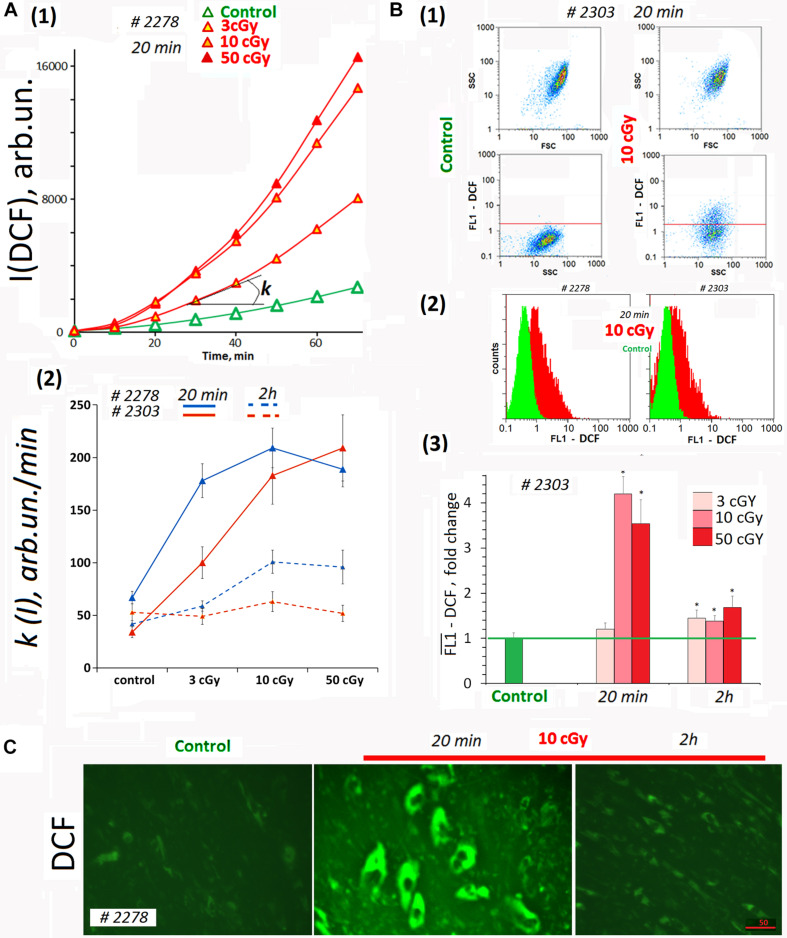
ROS levels in the irradiated cells. **(A)** Plate assay (FL-reader). The cells were analyzed using total fluorescence assay in the 96-well plate format at λex = 490 nm and λem = 524 nm. (1) Example of reaction rate constant determination for DCF formation when DCFH reacts with ROS. Cells were irradiated with doses of 3, 10, and 50 cGy and were cultivated for 20 min. The cultivation environment was replaced by 5 μM H_2_DCFH-DA in PBS solution and a fluorescence intensity increase was detected. The slope of the line is the reaction rate constant for DCF formation [*k(I)*]. (2) The constants *k(I)* of the rate of DCF formation in MSCs #2278 and #2303. The H_2_DCFH-DA reagent was added 20 min or 2 h after irradiation. Average values and SD are given for three experiments. In each experiment, cells were analyzed in four wells of the plate. **(B)** FACS. (1) Plots: SSC versus FSC and FL1-DCF versus SSC. (2) The distribution of MSCs #2278 and #2303 (10 cGy, 20 min after LDIR) with varying DCF signal. (3) Median signals for FL1–DCF. Signals are normalized to control values. Average values and SD are given for three experiments. *p < 0.001 (*U* test). **(C)** Fluorescence microscopy. Before the treatment with LDIR, cells were grown for 48 h in slide flasks. Twenty minutes or 2 h after LDIR, the cultivation environment was replaced by 5 μM H_2_DCFH-DA in PBS solution, and a fluorescence intensity increase was detected. The experiment was repeated three times.

Dichlorofluorescein (DCF) was detected on a plate reader to provide quantitation of total ROS in the cells and in the medium (Figure 2A). In the analysis, we did not take into account the autofluorescence signal. The DCF signal was two- to three-fold stronger than the autofluorescence (Figures 1A1, 2A1). Figure 2A1 shows the kinetics of the total fluorescent signal 20 min after MSC LDIR irradiation. Figure 2A2 shows the rate constants of the DCF formation reaction for two MSC lines. The analysis was performed 20 min and 2 h after irradiation of cells at a dose of 3, 10, or 50 cGy. Maximum DCF synthesis rates (maximum ROS) were observed for doses of 10 and 50 cGy 20 min after irradiation. Two hours later, the rate of DCF synthesis decreased two- to three-fold, indicating the ROS level decreased in the cell lines.

Average ROS amount in the cells was measured by FACS method. H2DCFH-DA reagent in PBS was added to the cells 20 min after LDIR exposure (Figure 2B). The radiation dose of 3 cGy did not lead to strong ROS synthesis in MSCs (Figure 2B3). At the same time, the dose of 10 or 50 cGy briefly increased the ROS level in MSCs several-fold in 20 min. The ROS level decreased 2 h after exposure to 50- or 10-cGy dose (Figure 2B3). However, after the dose of 50 cGy ROS level values may be underestimated, as under strong oxidative stress a part of the DCF may diffuse from the cells into the environment, because of the cell membrane damage due excessive ROS amount in the cell (Royall and Ischiropoulos, 1993).

Reactive oxygen species levels differences obtained by the two methods for the population MSCs (3 cGy) (Figures 2A2,B3) can be explained by ROS synthesis only on the cell surface and DCF reagent washing after cell separation for FACS. The plate reader allowed analyzing the overall DCF signal – both in cells and in the medium.

The data were confirmed by fluorescence microscopy. Figure 2C shows the result of the ROS level analysis in the living MSC (10cΓp) by fluorescence microscopy. In 20 min after the radiation, the ROS synthesis level increased, and in 2 h, it decreased.

Thus, after MSC irradiation with doses 10 or 50 cGy, we observed a short-term but significant ROS level increase in both cell lines (Figure 2A2). Apparently, 3-cGy dose stimulates ROS synthesis on MSCs surface only.

### LDIR Increases *NOX4* Expression

Radiation caused ionization of water and the formation of short-lived superactive radicals. ROS synthesis after radiation termination was associated with biological processes in the irradiated cell. Intensive irradiated MSC autofluorescence suggests that NADPH oxidase family (NOX) enzyme level in these cells increased. These proteins have been shown to contribute significantly to the MSC autofluorescence (Campbell et al., 2020).

Most NOX isoforms produce O_2_^∙⁣–^ as a primary product. However, H_2_O_2_ was the dominant ROS detected for NOX4. It has been reported that levels of NOX4 expression are higher than others NOXs in adipose-derived MSCs. Generation of ROS by NADPH oxidase NOX 4 appeared to be necessary for positive regulation of MSC proliferation and adipogenic differentiation (Park et al., 2011; Atashi et al., 2015).

**FIGURE 3:**
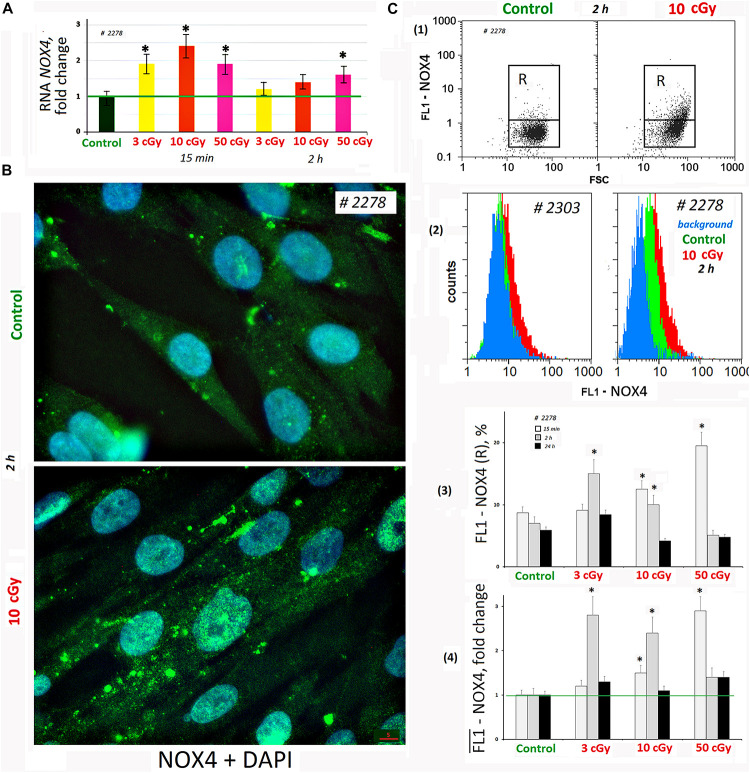
LDIR induce expression of *NOX4* in MSCs. **(A)** Quantitative reverse transcriptase–polymerase chain reaction. *NOX4* RNA levels changes in treated cells (15 min or 2 h after LDIR) compared to control. Average values and SD are given for three experiments. Reference gene *TBP*. *p < 0.001 (*U* test). **(B)** Fluorescence microscopy. Localization of FITC-labeled antibody against NOX4 in the irradiated MSC (10 cGy, 2 h after LDIR) and control MSC. **(C)** FACS. (1) Plots: FL1-NOX4 versus SSC. R: gated area, cells with an increased NOX4 expression. (2) The distribution of #2278 and #2303 MSCs (10 cGy, 2 h after LDIR) with varying FL1-NOX4 signal. To quantify the background fluorescence, we stained a portion of the cells with secondary FITC-conjugated antibodies only (blue color). (3) The content of the cells with an increased level of NOX4 expression (gate R). (4) Median signals for FL1–NOX4. Signals are normalized to control values. Average values and SD are given for three experiments. *p < 0.001 (*U* test).

We analyzed changes in the *NOX4* gene expression in response to LDIR (PNc.3). In 15 min after exposure to LDIR, *NOX4* expression increased in all the samples. Two hours later, *NOX4* expression decreased, remaining elevated after 50-cGy dose (Figure 3A).

Fluorescence-activated cell sorting confirms NOX4 protein increase after MSC irradiation. Both the content of cells with high protein levels (Figure 3C3) and the total amount of protein in the population increase (Figure 3C4). Maximum protein levels were observed in MSC (50 cGy) populations after 15 min and MSCs (3 or 10 cGy) after 2 h. After 24 h, the NOX4 level in the cells decreased to the control values. The MSCs differed significantly by the NOX4 level (Figure 3C2). Line #2278 contained large NOX4 amounts, both in the control and after irradiation.

Fluorescence microscopy data showed that in control cells, NOX4 was localized mainly in the cytoplasm and on the cell membrane (Figure 3B). The NOX4 level increased significantly in irradiated MSCs. Moreover, NOX4 expression increased not only in the cytoplasm, but also in the cell nuclei.

### LDIR Change the Antioxidant Response

Oxidative stress caused by an increase of ROS production can activate an antioxidant response in which one of the main regulators is NRF2. Emerging research has identified Nrf2 as a critical factor for promoting survival of mammalian cells subjected to IR. Nrf2 accumulates in the cytoplasm, and after phosphorylation, pNrf2(Ser40) translocates into the nucleus where it activates transcription of antioxidant response elements (Kaspar et al., 2009; Chen et al., 2015; Sekhar and Freeman, 2015; Sies and Feinendegen, 2017).

Low-dose ionizing radiation did not significantly affect NRF2 level in the MSCs (Supplementary Figure 2A). We observed a 30% increase in the amount of NRF2 in the population #2278 MSCs (10 cGy) 15 min after irradiation. However, 2 h after irradiation, the amount of protein decreased. The average amount of NRF2 increased in the MSC (50 cGy) after 24 h. For cells of the #2303 MSC line (10 cGy) that were cultured 15 min, 30 min, 1 h, and 2 h after irradiation, we found a negative correlation between NOX4 and NRF2 content (Supplementary Figure 2B).

**FIGURE 4:**
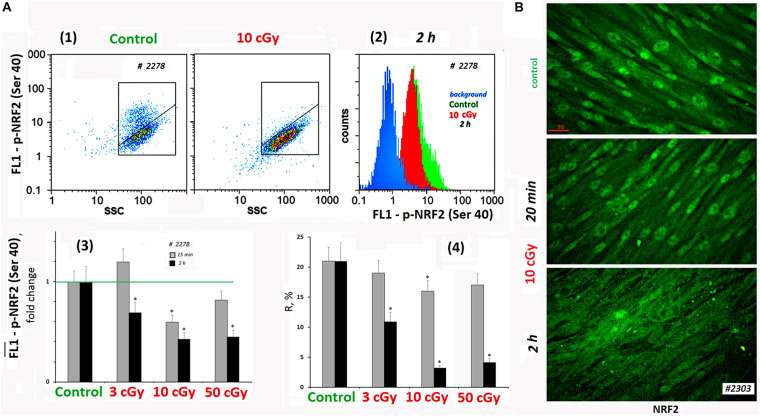
LDIR block activity of NRF2 in MSCs. **(A)** FACS. (1) Plots: FL1–p-NRF2(Ser40) versus SSC. R: gated area, cells with an increased level of p-NRF2(Ser40) expression. (2) The distribution of the MSCs (10 cGy, 2 h after LDIR) with varying FL1–p-NRF2(Ser 40) signal. To quantify the background fluorescence, we stained a portion of the cells with secondary FITC-conjugated antibodies only (blue color). (3) The content of the cells with an increased p-NRF2(Ser40) expression (gate R). (4) Median signals for FL1–p-NRF2 (Ser40). Average values and SD are given for three experiments. *p < 0.001 (*U* test). The time of cell cultivation after LDIR is shown in the figure. **(B)** Fluorescence microscopy. Localization of FITC-labeled antibody against NRF2 in the irradiated MSCs (10 cGy, 20 min or 2 h after LDIR) and control MSC. In the control MSCs and in the MSCs (10 cGy, 20 min), the protein NRF2 is located in the nucleus. In the MSCs (10 cGy, 2 h), the protein is located outside the nucleus.

The activity of NRF2 transcription factor was determined by its phosphorylation [p-NRF2(Ser40)] and by the localization of the protein in the cell (Boo, 2020). Figure 4A shows FACS data illustrating p-NRF2(Ser40) changes in MSCs (10 cGy) after 15 min and 2 h. In control MSCs, we observed a high level of NRF2 phosphorylation in 20% of the cells. Exposure decreased average population p-NRF2 (Ser40) level, NRF2 phosphorylation more pronounced 2 h after exposure.

According to the microscopy data, the transcription factor NRF2 in the control conditions was localized in the MSCs nucleus, reflecting the active antioxidant protection. In population #2303 MSCs (10 cGy), the level and localization of NRF2 did not change significantly 20 min after irradiation (Figure 4B). Two hours later, the NRF2 expression in the cell was almost unchanged, but we observed NRF2 factor migration from the nucleus to the cytoplasm in most cells. This fact confirms the antioxidant activity decrease in MSCs after low-dose irradiation.

### LDIR Cause Nuclear DNA Damage

#### Peculiarities of the Irradiated MSCs Analysis

We analyzed three types of DNA damage in irradiated MSCs: (1) DNA oxidation (marker – 8-oxodG, FACS method), (2) SSBs and DSBs (method of nuclear electrophoresis in alkaline gel), and (3) DSBs only (marker – phosphorylated form of histone H2AX, FACS method).

Using all three methods, we encountered a problem complicating the analysis of irradiated cells: MSCs (50 cGy) were found to be more sensitive to the cell sample preparation procedures than MSCs (C), MSCs (3 cGy), or MSCs (10 cGy). During a usual isolation and fixation procedure, which included cell washing, treatment with trypsin, EDTA, and centrifugation, damaged cells were lost (detached from the carrier, partially destroyed, and worse separated by a centrifugation). As a result, the least damaged cells were selected. In a number of experiments, we received paradoxical results – the level of MSC damage (50 cGy) was lower than the level of MSC damage (3 cGy). Therefore, the procedure for obtaining samples has been changed (see section “Materials and Methods”).

#### Cellular DNA Oxidation (8-oxodG Content)

**FIGURE 5:**
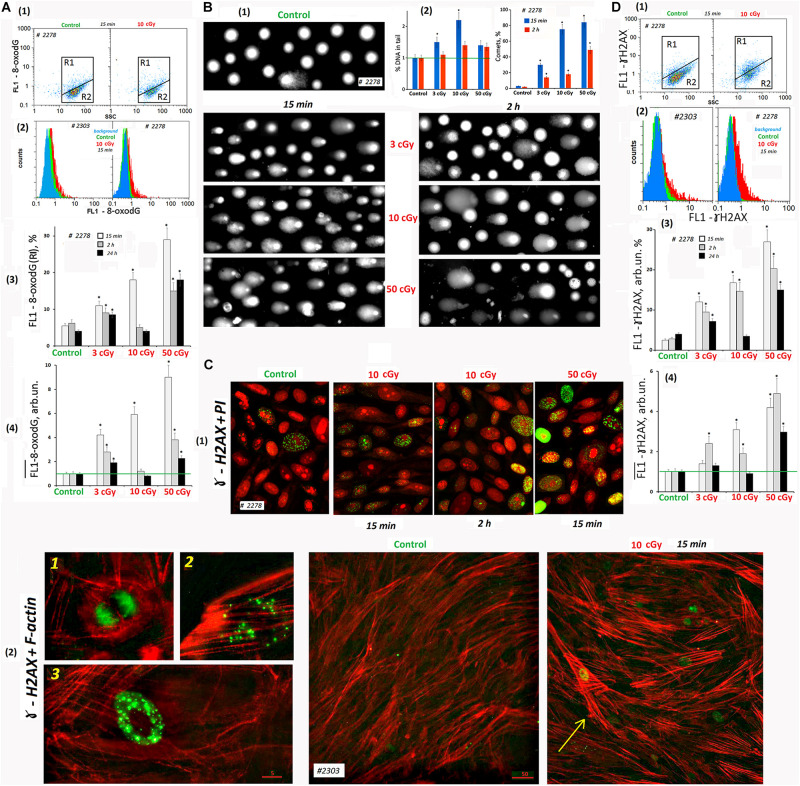
LDIR induce DNA damage and DNA breaks in MSCs. **(A)** FACS. Cell DNA oxidation (marker-8-oxodG). (1) Plots: FL1- 8-oxodG versus SSC. R1: gated area, cells with an increased level of 8-oxodG. (2) The distribution of #2278 and #2303 MSCs (10 cGy, 15 min after LDIR) with varying FL1–8-oxodG signal. Background fluorescence (blue color): the cells were stained with secondary FITC-conjugated antibodies only. (3) The content of the cells with an increased 8-oxodG (R1) level. (4) Median signals for FL1–8-oxodG. Average values and SD are given for three experiments. *p < 0.001 (*U* test). The time of cell cultivation after LDIR is shown in the figure. **(B)** Comet assay (alkaline agarose gel electrophoresis, stained with PI). Determination of the total DNA breaks level. (1) Gallery of comets in different cell populations. The photograph provides data from several images of comets gels. The cultivation times and radiation doses are shown in the figure. (2) Relative frequency of DNA percent distribution in the comet tail (left graph) and the number of comets in the cell populations (right graph). **(C)** Fluorescence microscopy. Determination of DSBs (ɣ-H2AX foci) level. (1) Gallery of photographs of cell nuclei (PI – red) with ɣ-foci (green) for different cell populations. The photograph provides the data from several images. The cultivation times and radiation doses are shown in the figure. (2) Photographs of the cells with ɣ-foci (green) for MSCs (control) and MSCs (10 cGy) populations. Rhodamine phalloidin was used to label cytoskeletal F-actin. 1, 2, and 3: Examples of the cells containing ɣ-foci. An arrow indicates the cell with DNA damage and increased F-actin levels. **(D)** FACS. Determination of DSBs (ɣ-H2AX foci) level. (1) Plots: FL1- ɣ-H2AX versus SSC. R1: gated area, cells with an increased ɣ-H2AX level. (2) The distribution of #2278 and #2303 MSCs (10 cGy, 15 min after LDIR) with varying FL1-ɣ-H2AX signal. Background fluorescence (blue color): the cells were stained with secondary FITC-conjugated antibodies only. (3) The content of the cells with an increased level of ɣ-H2AX (gate R1). (4) Median signals for FL1-ɣ-H2AX. Average values and SD are given for three experiments. *p < 0.001 (*U* test). The time of cell cultivation after LDIR is shown in the figure.

Low-dose ionizing radiation increased ROS production leading to the nuclear DNA oxidation. FITC-labeled antibodies were used for 8-oxodG detection. FACS method revealed the presence of two populations of the control cells – with high 8-oxodG level: 3–8% from the entire cell population (Figure 5A, gate R1) and with low 8-oxodG level (R2). The number of cells (R1) increased in 15 min after radiation doses of 3, 10, and 50 cGy two-, three-, and five-fold, respectively (Figure 5A3). The total 8-oxodG level in the population of irradiated cells (medians of signal values, Figure 5A4) increased several-fold 15 min after irradiation.

Two hours after radiation exposure, 8-oxodG fluorescence level decreased, and the maximum response was observed for MSCs (10 cGy). A day after exposure, 8-oxodG levels in the populations of MSCs (3 cGy) and MSCs (50 cGy) remained above the control level, in contrast to MSCs (10 cGy) (Figure 5A3,4).

#### SSBs and DSBs in the Cell DNA (Comet Assay)

DNA damage in individual cells nuclei was measured using the comet assay. It detects both SSBs and DSBs. The studied cells were lysed and then subjected to alkaline electrophoresis in an agarose gel stained with PI. The DNA damage (thread breaks) was estimated as the percentage of fluorescent DNA in the comet’s tail. In 15 min after radiation was applied in 3-, 10-, and 50-cGy doses, the DNA percentage in the comet’s tail increased by 40, 220, and 30%, respectively (Figure 5B2). Two hours after irradiation with a dose of 3 cGy, the percentage of DNA in the tail was reduced to the control values. After doses of 10 cGy and 50 cGy, the percentage of DNA in the comet tail remained increased by 30% compared to the control.

To explain the seemingly reduced DNA breaks number in the MSC population (50 cGy) compared to MSCs (10 cGy), we performed a visual comets analysis. Figure 5B1 shows examples of comets obtained by summation of the several gels photographs. Photographs of gels obtained for MSCs (50 cGy) differed significantly from the rest by the presence of individual chromatin fragments and weakly colored comets that have lost some of their DNA. In general, the cell nuclei of the population MSCs (50 cGy) were more destroyed than the nuclei of MSCs (10 cGy). This fact indicates significantly greater cell damage in the MSC population (50 cGy) and correlates with data on the loss of some cells in the MSC population (50 cGy) during the standard procedure for these cells’ isolation. The total cell population damage can be characterized by the number of comets per 100 nuclei (Figure 5B2). The population of MSCs (50 cGy) contained the maximum number of comets, both 15 min and 2 h after irradiation.

#### DSBs in the Cell DNA (ɣH2AX Foci)

Double-stranded DNA breaks may be detected using histone protein involved in the DNA chromatin packaging (H2AX) that is phosphorylated at the DNA break site by the serine residue 139. As a result, phosphorylated histones associated with labeled antibodies, called ɣH2AX foci, were visualized in the cell nucleus.

Therefore, the cells were fixed with formaldehyde and stained with FITC-labeled γH2AX antibodies and analyzed by cytofluorometry. Two cellular subpopulations were identified according to fluorescence level: with high signal level (R1) and the main MSC fraction R2 with low fluorescence (Figure 5D). In the control group, the average R1 fraction was 3–6% of the total population. After 15 min of radiation exposure, the number of such cells increased to 12, 17, and 28% for cells irradiated with 3, 10, or 50 cGy, respectively (Figure 5D3). The overall γH2AX level (median of signal values) also increased proportionally to the radiation dose. A day after MSC irradiation, we observed a γH2AX decrease to the control level in the MSCs (3 cGy) and MSCs (10 cGy) populations. The marker level in the MSC (50 cGy) remained higher than the control.

Fluorescence microscopy and FACS provided similar results in the analysis of LDIR effects on the ɣH2AX content in the MSC population (Figure 5Ñ). During the analysis, we took precautions to preserve maximum possible number of the cells on the carrier. Two variants of cell staining were used: PI staining of the nuclei (Figure 5C1) and polymer F-actin staining (Figure 5C2). In MSCs, PI allowed localizing the nucleoli (Konkova et al., 2020). The F-actin reagent allowed tracing the stress fibers formation in response to ROS after LDIR (Ermakov et al., 2011).

Mesenchymal stem cells (C) contained a small number of cells with multiple foci (about 2%, photo 3 in Figure 5C2) and cells with a small number of foci (about 3%, photo 2). In addition, there were cells in mitosis phase that were also stained with antibodies to γH2AX (photo 1, Figure 5C2).

Fifteen minutes after irradiation, the number of cell types 2 and 3 increased. In the population of MSCs (10 cGy), after 2 h, most cells remained with a small number of gamma foci. In the MSC (50 cGy), after 2 h, there were many cells with a high ɣH2AX content (Figure 5C1). Apparently, we lost these cells during the standard cell isolation and fixation procedure for FACS. It is interesting to note that ɣH2AX foci were localized in irradiated cells outside the nucleolus or on the surface of the nucleolus, but not inside the nucleolus structure.

Mesenchymal stem cell irradiation induced the formation of F-actin stress fibers (Figure 5C2, shown by the arrow). The highest level of F-actin was observed in the cells whose nuclei contain ɣH2AX foci. Apparently, these cells were mostly affected by ROS. We observed approximately the same response of two MSC populations to a dose of 50 cGy. However, the MSCs #2303 line responded to the 10-cGy dose with a lower ɣH2AX expression (Figure 5D2).

### LDIR Causes Cell Cycle Arrest

It is known that oxidative stress, causing the chromatin damage, inhibits cell division at all cell cycle stages and blocks the proliferation. We analyzed the LDIR effect on the proliferating cell number in MSCs using FACS method and antibodies to the KI-67 proliferation marker and PCNA. The cells were stained with PI to determine the DNA amount.

**FIGURE 6:**
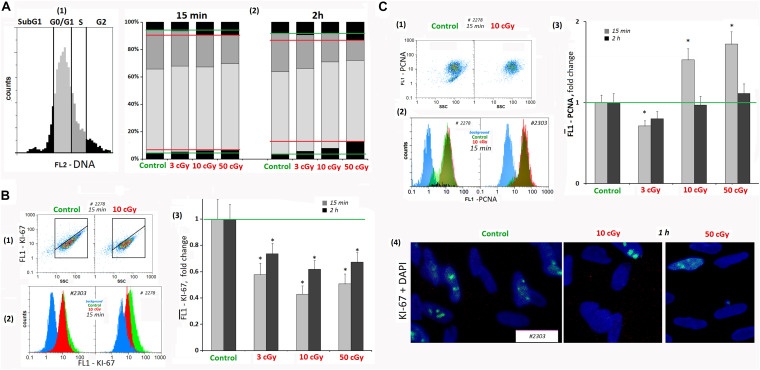
LDIR induce cell cycle arrest in MSCs. **(A)** FACS. The DNA amount in the MSCs (cell nuclei staining with PI). (1 and 2) MSC fractions with the DNA amount corresponding to the Sub G1, G0/G1 -, S -, and G2/M cell cycle phases. The cultivation times and radiation doses are shown in the figure. **(B)** FACS (1–3). Cell nuclei staining with FITC-labeled antibody against KI-67. (1) Plots: FL1- KI-67 versus SSC. (2) The distribution of the MSCs (10 cGy, 15 min after LDIR) with varying FL1–KI-67 signal. (3) Median signals for FL1–KI-67. (4) Fluorescence microscopy. Localization of FITC-labeled antibody against KI-67 in the irradiated MSC (10 and 50 cGy) and MSC **(C)**. Average values and SD are given for three experiments. *p < 0.001 (*U* test). **(C)** FACS. Cell nuclei staining with FITC-labeled antibody against PCN (A). (1) Plots: FL1- PCNA versus SSC. (2) The distribution of the MSCs (10 cGy) with varying FL1–PCNA signal. (3) Median PCNA signals.

Analyzing MSCs for the KI-67 marker content, we found that when radiation doses of 3, 10, and 50 cGy were applied, KI-67 expression level decreased by 40–50% in 15 min and increased 2 h after the radiation, remaining reduced (Figure 6B1–4).

The dose of 3 cGy reduced MSC PCNA levels by 15–20%. However, after LDIR (10 and 50 cGy) PCNA protein expression differed from KI-67 protein expression. In 15 min after 10- and 50-cGy doses, a 40–60% PCNA level increase was observed (Figure 6C). As PCNA is a delta polymerase transcription factor also participating in DNA repair (Shivji et al., 1992), the observed differences in the low radiation dose effects may be explained by the repair process induction with proliferative activity decrease as a result of the cell cycle arrest.

Mesenchymal stem cell culture at the subconfluent stage (∼70%) included the cells with different DNA amount, which may be classified into four groups (Figure 6A): subG1, G0/G1, S, and G2/M. In irradiated MSCs, the number of cells in the G1 and G2/M phases increased in 15 min; i.e., G1 and G2/M cell cycle arrest was observed, which is especially pronounced after 50-cGy dose.

Cyclin D1 is a protein that specifically regulates the G1/S phase transition in the cell cycle. After LDIR (15 min), the CCND1 gene expression in MSCs was reduced by 30–40%, and the CDKN2 and CDKN1A gene expression increased by 50–100%, which also reflects a short-term cell cycle stop. However, 2 h after low radiation doses, the CCND1 expression in MSCs increased by 10–20% compared to the control, and the CDKN2 and CDKN1A gene expression decreased, reaching the control values (Table 1).

**TABLE 1:** Dependence of the changes in the levels of genes *CCND1*, *CDKN2*, and *CDKN1A* in irradiated MSCs on the time after exposure (RT-PCR).

**Gene**	**Expression levels, fold change**			
	**Time**	**3 cGy**	**10 cGy**	**10 cGy**
*CCND1*	30 min	0.7 ± 0.2	0.6 ± 0.2*	0.8 ± 0.3
	2 h	1.3 ± 0.4	1.7 ± 0.2*	1.4 ± 0.2*
	24 h	1.1 ± 0.3	1.4 ± 0.2*	1.1 ± 0.3
*CDKN2*	30 min	1.5 ± 0.2*	2.1 ± 0.3*	1.8 ± 0.3*
	2 h	1.1 ± 0.2	0.9 ± 0.1	1.1 ± 0.3
	24 h	1.0 ± 0.3	0.8 ± 0.2	0.9 ± 0.2
*CDKN1A*	30 min	1.4 ± 0.2*	2.2 ± 0.2*	1.7 ± 0.2*
	2 h	1.2 ± 0.3	0.9 ± 0.2	1.1 ± 0.3
	24 h	0.9 ± 0.2	0.8 ± 0.3	1.0 ± 0.3

mRNA level – average expression of genes in treated MSCs compared to control (for three biological replicates). Reference gene – *TBP*. *p < 0.001, nonparametric *U* test (qRT-PCR).

### LDIR Activates Nuclear DNA Repair

Nuclear DNA damage after oxidative stress induced by the LDIR activates signaling cascades that control DNA repair. Double-strand DNA breaks are among the most dangerous DNA damage forms. BRCA1 and BRCA2 are unrelated proteins, but both repair damaged DNA or destroy the cells if DNA cannot be repaired.

**FIGURE 7:**
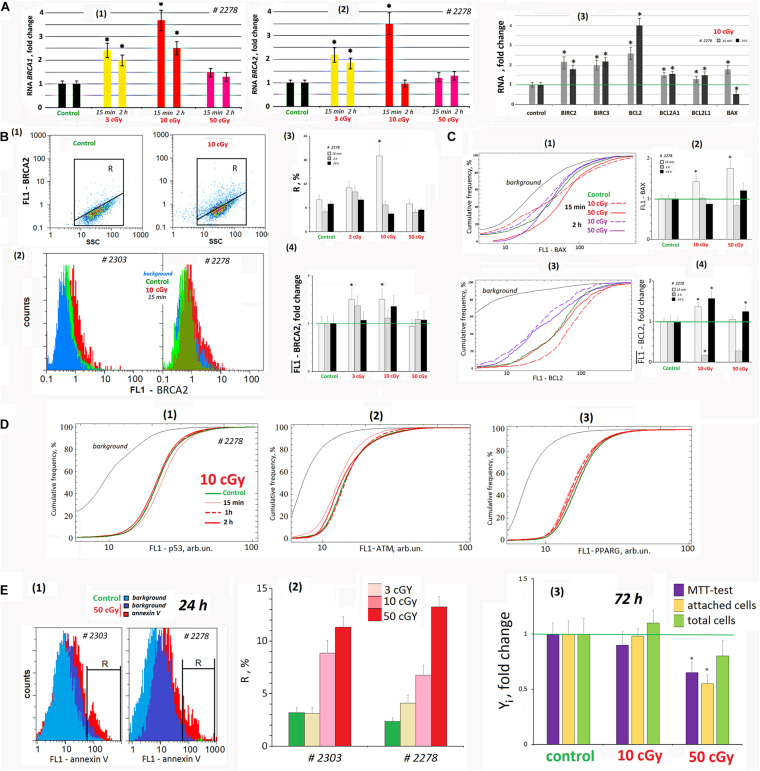
LDIR affect the processes of apoptosis and repair in the cells. **(A)** Quantitative reverse transcriptase–polymerase chain reaction. (1) and (2) Changes in the levels of RNA *BRCA1* and RNA *BRCA2* in LDIR-treated cells compared to control (three biological replicates). The time of cell cultivation after LDIR is shown in the figure. (3) Proapoptotic gene *BAX 1* RNA level and BIRC and BCL families antiapoptotic genes changes in the MSCs (10 cGy) compared to control (three biological replicates). Reference gene *TBP*. *p < 0.001 (*U* test). **(B)** FACS. BRCA2 protein level changes. (1) Plots: FL1- BRCA2 versus SSC. R: gated area, cells with an increased BRCA2 level. (2) MSCs (10 cGy, 15 min after LDIR) with varying FL1–BRCA2 signal distribution. (3) The content of the cells with an increased BRCA2 level. (4) Median FL1–BRCA2 signals. Average values and SD are given for three experiments. *p < 0.001 (*U* test). The time of cell cultivation after LDIR is shown in the figure. **(C)** FACS. (1) The cumulative distribution of the MSCs (10 and 50 cGy) with varying FL1–BAX1 signal. (2) Median signals for FL1–BAX1. (3) The cumulative distribution of the MSCs (10 and 50 cGy) with varying FL1–BCL2 signal. (4). Median signals for FL1–BCL2. The cultivation times and radiation doses are shown in the figure. Average values and SD are given for three experiments. *p < 0.001 (*U* test). **(D)** FACS. (1–3) The cumulative distribution of the MSCs (10 cGy) with varying FL1–p53, FL1-ATM, and FL1–PPARG signals. The time of cell cultivation after LDIR is shown in the figure. **(E)** FACS (1, 2). (1) The distribution of the MSCs (50 cGy) with varying FL1–annexin V. R: gated area, cells with an increased annexin V level. (2) The content of the cells with an increased annexin V level. (3) Evaluation of cell viability 72 h after irradiation. MTT test, the number of the attached cells, and the total cell number. Parameter values are normalized to the control values.

Fifteen minutes after LDIR (3 and 10 cGy) were applied on the MSCs, the *BRCA1* gene expression increased and remained elevated 2 h later (Figure 7A1). Similarly, *BRCA2* gene expression in 15 min after radiation dose of 3 and 10 cGy increased by 120 and 250% (Figure 7A2). Two hours after the 3-cGy radiation dose, the BRCA2 expression remained increased by 80%. Under 10-cGy dose, it decreased to the control values. The 50-cGy dose produced no significant effect on the *BRCA1* and *BRCA2* expression.

The gene expression changes were confirmed by the protein BRCA2 synthesis levels. Fifteen minutes after 3- and 10-cGy doses, the BRCA2 level increased (Figure 7B). Two hours after the 3-cGy dose, the *BRCA2* gene expression remained elevated. After the 10-cGy dose, it decreased to the control values. The 50-cGy dose produced no significant effect on the BRCA2 expression.

We analyzed in the population of MSCs (10 cGy) the content of three transcription factors regulating stem cell response to radiation – p53, ATM, and PPARG (Figure 7D). It has been shown that the early MSC (10 cGy) response is not accompanied by these factors’ increase in the cells.

### LDIR Changes the Apoptosis Level

We analyzed the RNA levels of genes involved in proapoptotic and antiapoptotic response of MSCs to LDIR (Figure 7A3). After 15 min, the expression of the proapoptotic BAX1 gene and the antiapoptotic BIRC and BCL genes significantly increased in the MSC population (10 cGy). Expression of all the genes except BAX1 in irradiated MSCs remained elevated after 24 h.

Using the FACS method, we analyzed BAX1 and BCL2 proteins levels in the MSC (10 cGy) and (50 cGy) populations. Fifteen minutes after irradiation, BAX1 level changes in the cells. In MSC (10 cGy), half of the cells contain elevated BAX1 level, whereas the remaining cells reduce BAX1 content. In the MSC (50 cGy), the protein increased in all the cells (Figure 7C1). However, after 2 h, the BAX1 level in MSCs (10 cGy) decreased to the control level. In the MSC population (50 cGy), only 20% of cells after 2 h contain increased protein levels compared to control.

The level of antiapoptotic protein BCL2 increased after 15 min in MSC population (10 cGy) and slightly increased in 30% of the cells in the MSC (50 cGy) (Figure 7C3). BCL2 levels drop 2 h after exposure and rise again 24 h later.

We determined the content of cells with signs of apoptosis in MSCs irradiated with doses of 3, 10, and 50 cGy. For the analysis, we took into account the residual background signal reflecting the autofluorescence of unfixed cells. For the population of MSK #2278 (50 cGy), this signal remained high enough after 24 h (Figure 7E1). Analysis of the phosphatidyl-serine level (annexin V marker) in irradiated MSCs at shorter times (2 h and 15 min) was practically impossible, as the autofluorescence signal significantly exceeded the annexin V signal. 24 h after irradiation, the number of apoptotic cells increased to a maximum of 10–14% compared to the control (about 4% of cells). The maximum number of cells with signs of apoptosis was observed for MSC populations (50 cGy).

Figure 7E3 shows the data on the state of MSC (10 cGy) and MSC (50 cGy) populations 3 days after exposure. A standard MTT test recorded a decrease in the metabolic activity of irradiated cells. However, this result does not reflect the real picture, as we found the same decrease in the number of cells attached to the carrier. Apparently, some of the irradiated cells were lost during washing and cell fixation procedures during the MTT test. Analysis of the total number of cells, taking into account the cells that remained in the washing solutions, did not find significant differences in the number of irradiated cells and the control. There was a tendency to increase the number of cells in the MSC population (10 cGy) and decrease in the MSC population (50 cGy), but the differences with the control were nonsignificant.

## Discussion

**FIGURE 8:**
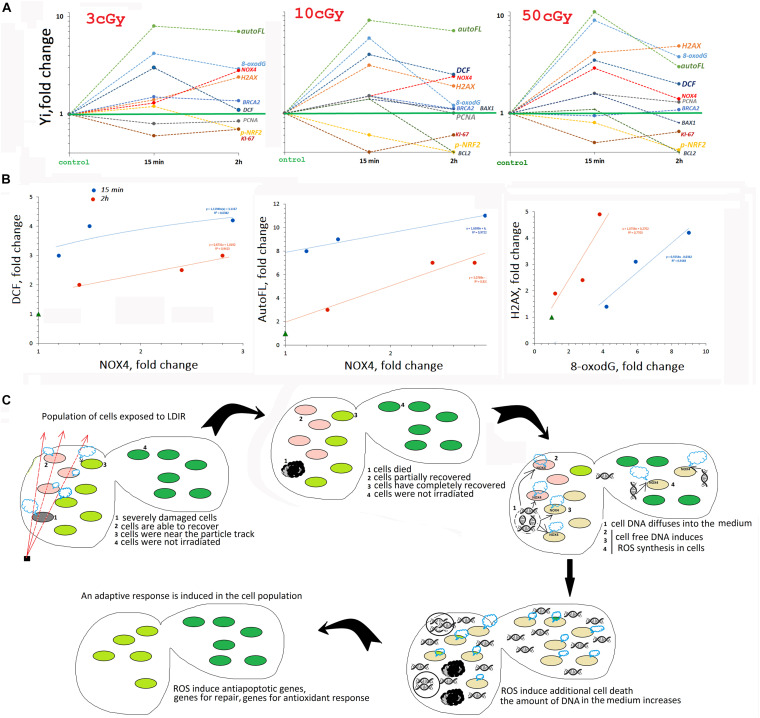
**(A)** The summarized data obtained in the study for line #2278. Mainly FACS data are given. **(B)** Analysis of the dependence of ROS (DCF) and auto FL1 changes on NOX4 change and the dependence of ɣ-H2AX level on 8-oxodH level. **(C)** The scheme describing MSC population response to LDIR.

We analyzed the early response of the two MSC lines from human adipose tissue to the action of LDIR (3, 10, and 50 cGy). Both lines showed a qualitatively similar response to LDIR, with some effects differing quantitatively. Figure 8A summarizes the data obtained for line #2278.

### Early MSC Response to LDIR Includes Transient Oxidative Stress

The period from 15 min to 2 h after MSC irradiation is characterized by an increased ROS level. Maximum ROS levels are recorded 15 min after exposure to 10- and 50-cGy doses and are reduced after 2 h.

An increase in the ROS level induces autofluorescence in MSCs. Previously, the authors reported that many different human and murine cell types respond to IR with a striking rise in autofluorescence that is dependent on dose and time (Schaue et al., 2012). Autofluorescence increased upon irradiation of the cells with radiation doses of 50 cGy and more. MSCs were found to be more sensitive. Autofluorescence in these cells increased even when exposed to small doses of radiation (Figure 1).

The high ROS level could be explained by the production of secondary, less active, and longer-living ROS as a result of the reaction of superactive radicals with the medium and cellular components. However, we found that ROS levels positively correlated with the NOX4 protein level, catalyzing hydrogen peroxide formation (Figure 8B). *NOX4* gene expression in irradiated cells increased both at the transcription and at the protein level (Figure 3). The protein was localized on the cell membrane, in the cytoplasm, and in the nuclei of irradiated cells. Therefore, NOX4 can produce hydrogen peroxide in all cell structures. At the same time, the activity of the factor NRF2, which controls the antioxidant cellular response, was blocked in the irradiated cells during the same time period (Figure 4). The total NRF2 amount in irradiated cells changed not very significantly, but the protein was not active, as evidenced by its migration from the nucleus to the cytoplasm and a decrease of its phosphorylated form, associated with NRF2 activity.

Thus, the early MSC response to LDIR suggests short-term maintenance of a certain ROS level by pro-oxidant systems activation and antioxidant response inhibition. This conclusion, at first glance, seems paradoxical. IR increased the ROS level due to the process of ionization of environmental components and cells. However, to develop a response to low radiation doses, the cell needs to additionally maintain the ROS level at a certain level. It can also be assumed that the response to LDIR requires ROS of a specific chemical structure, such as hydrogen peroxide or its derivatives.

The endogenous ROS role in the MSC response to LDIR can be clarified by considering the main components of the response of a cell population to radiation stress. Four well-known main processes occur simultaneously in an irradiated cells population:

(1) DNA damage repair in cells that were affected by the particle passage or by superactive short-living radicals at the time of irradiation.
(2) Elimination of cells with multiple unrepaired lesions from the population (apoptosis).
(3) Adaptive response development that increased the irradiated cells resistance to the repeated radiation exposure.
(4) Bystander effect development that may induce adaptive response similar to irradiated cells in nonirradiated cells of the population.

### LDIR Damage Cellular DNA

Low-dose ionizing radiation seriously damaged the DNA of cells. Immediately after irradiation, an increase in the oxidation marker 8-oxodG level was detected in the population (Figure 5), which was proportional to the radiation dose (Figure 8). At the same time, double-strand breaks were produced (the level of ɣH2AX increased), in a number proportional to the 8-oxodG level. The total number of cells with DNA breaks increased significantly. In response to DNA damage, the cell cycle was blocked, and the level of the PCNA protein, which was involved in excision DNA repair, increased (Figure 6). In addition, in the MSC populations (3 and 10 cGy), the expression of genes involved in DNA repair (BRCA1 and BRCA2) increased. DNA damage decreased after 2 and 24 h, with the most effective reduction observed in the MSC population (10 cGy). Apparently, the 10-cGy dose most actively induces repair processes in the cells.

### Early MSC Response to LDIR Includes the Radiosensitive Cell Fraction Apoptosis

The expression of the proapoptotic *BAX1* gene increased briefly in the MSC population (10 cGy) in parallel with the repair processes after 15 min (Figure 7A3). Moreover, in the MSC population (10 cGy), BAX1 protein amount in half of the cells was increased, and in the remaining cells, the amount of protein was reduced compared to the control. After 2 h, the expression of the *BAX1* gene decreased below control, but the amount of protein did not decrease. Along with the increased expression of the proapoptotic gene in the MSC population (10 cGy), the expression of the antiapoptotic genes of the *BIRC* and *BCL* family increased (Figure 7A3). The level of BCL2 protein increased in the first minutes after irradiation, but then decreased significantly. Thus, the first 2 h of irradiated cells cultivation was characterized by an increased BAX1/BCL2 ratio, indicating the irradiated cells’ apoptosis intensification. Most likely, cells with a high oxidation level and DNA breaks died first.

Unfortunately, the high background fluorescence level of irradiated cells did not allow using the annexin V protein for estimation of the number of cells in the MSC population with signs of apoptosis. However, such an assessment can be made by DNA staining with PI to analyze the number of hypodiploid cells (Figure 6A). The fluorescence level of PI in complex with DNA was several-fold stronger than the autoFL2 background signal. The SubG1 fraction size in the irradiated MSC populations in 2 h increased from 3 to 5–10%.

We believe that the transient ROS level increased the loss of a small fraction of radiosensitive cells after cell population irradiation by LDIR. This process is important for induction of two processes in irradiated population of cells – adaptive response and bystander effect. The bystander effect in a population of cells exposed to low doses suggests a mechanism for an adaptive response induction in cells not affected by radiation. Previously, it was shown that ROS, associated with increased NOX 4 expression, are necessary for the development of both an adaptive response and the bystander effect (Ermakov et al., 2009; Murley et al., 2018). ROS blocking inhibits the prosurvival adaptive response development. In a previous study, we also showed that conformational changes in the spatial chromatin organization, necessary for an adaptive response and the bystander effect induction in irradiated cells, are also blocked when ROS and the caspase 3 are blocked (Ermakov et al., 2009).

### A Hypothetical Scheme Describing the MSC Population Response to LDIR

In a number of previous studies, we have shown that cell-free oxidized DNA, appearing in the extracellular environment due to apoptosis of a small fraction of irradiated cells with severely damaged DNA, is an important factor ensuring an adaptive response development in the entire cell population, even in the part of the cells not exposed to radiation. Oxidized DNA, interacting with the surface of nonirradiated cells, induces the same cellular response as small radiation doses. First, oxidized DNA induces short-term ROS synthesis in cells, increasing NOX4 level. ROS level elevation activates the repair, antioxidative, and antiapoptotic systems, increasing the radiation resistance of the cells (Ermakov et al., 2011, 2013; Kostyuk et al., 2012, 2013, 2018; Loseva et al., 2012; Konkova et al., 2019).

Thus, we can assume that the transient oxidative stress, detected in MSCs exposed to LDIR, is necessary for the induction of the death of a small fraction of cells with severely damaged DNA in the first hours after irradiation procedure (see the diagram in Figure 8C). The oxidized DNA of these cells enters the extracellular environment. Further, this DNA fragments diffuse in the area with intact cells. Binding to receptors on the cell surface induces short-term ROS synthesis and the development of an adaptive response.

Obviously, it is almost impossible to consider any stage separately in the MSC response to the LDIR. The entire cell population responds to LDIR. Individual cellular subpopulations perform different functions. The most damaged cells in the population die, being the source of oxidized DNA fragments transmitting information about radiation exposure over long distances to intact cells in the population. Previously, the use of antioxidants (Ko et al., 2012) was proposed to increase stem cell survival under irradiation. However, the use of antioxidants immediately after irradiation can inhibit the adaptive response and increase cell death in the population upon repeated radiation exposure.

## Data Availability Statement

The authors acknowledge that the data presented in this study must be deposited and made publicly available in an acceptable repository, prior to publication. Frontiers cannot accept a manuscript that does not adhere to our open data policies.

## Ethics Statement

The study design was reviewed and approved by the Local Ethics Committee of FSBI “RSMG” (Federal State Budgetary Institution “Research Centre for Medical Genetics”) to meet the requirements of the Helsinki Declaration of 1975 as revised in 2013. An informed consent for the use of the surgical material had been obtained from each patient, from whom an anonymous cell culture was derived.

## Author Contributions

MK, SKo, SKu, VI, and NV designed the study. EE, MA, AK, and OD performed the experiments. NV performed the statistical analysis. SKo, NV, and PU wrote the initial draft and translated the manuscript to English. All authors participated in critical revision and approved the manuscript before submission.

## Conflict of Interest

The authors declare that the research was conducted in the absence of any commercial or financial relationships that could be construed as a potential conflict of interest.

## Footnotes

1 https://doi.org/10.1371/journal.pone.0077469

2 https://doi.org/10.1371/journal.pone.0189826

3 http://www.analystsoft.com/

## Supplementary Material

Supplementary Figure 1 **(A)** Autofluorescence of the irradiated MSCs (10 cGy) #2278. Photographs were taken with various fluorescent filters. **(B)** FACS. UVa irradiation effect on the MSCs and HSFs auto- fluorescence.

Supplementary Figure 2 **(A)** FACS. (1) Plots: FL1-NRF2 versus SSC. R: gated area, cells with an increased NRF2 expression. (2) The distribution of the MSCs (10 cGy) with varying NRF2 signal. (3) The content of the cells with an increased NRF2 expression. (4) Median signals for FL1 – NRF2. Average values and SD are given for three experiments. ^∗^*p* < 0.001 (*U* test). **(B)** Fluorescence microscopy. The dependence of FL1- NRF2 on FL1 – NOX4 changes in #2303 MSC (10 cGy).
