# Supplementary figures and images for: Mesenchymal Stem Cells Early Response to Low-Dose Ionizing Radiation
Source: Front Cell Dev Biol. 2020 Dec 14;8:584497. doi: 10.3389/fcell.2020.584497 (PMC7767887; doi:10.3389/fcell.2020.584497)

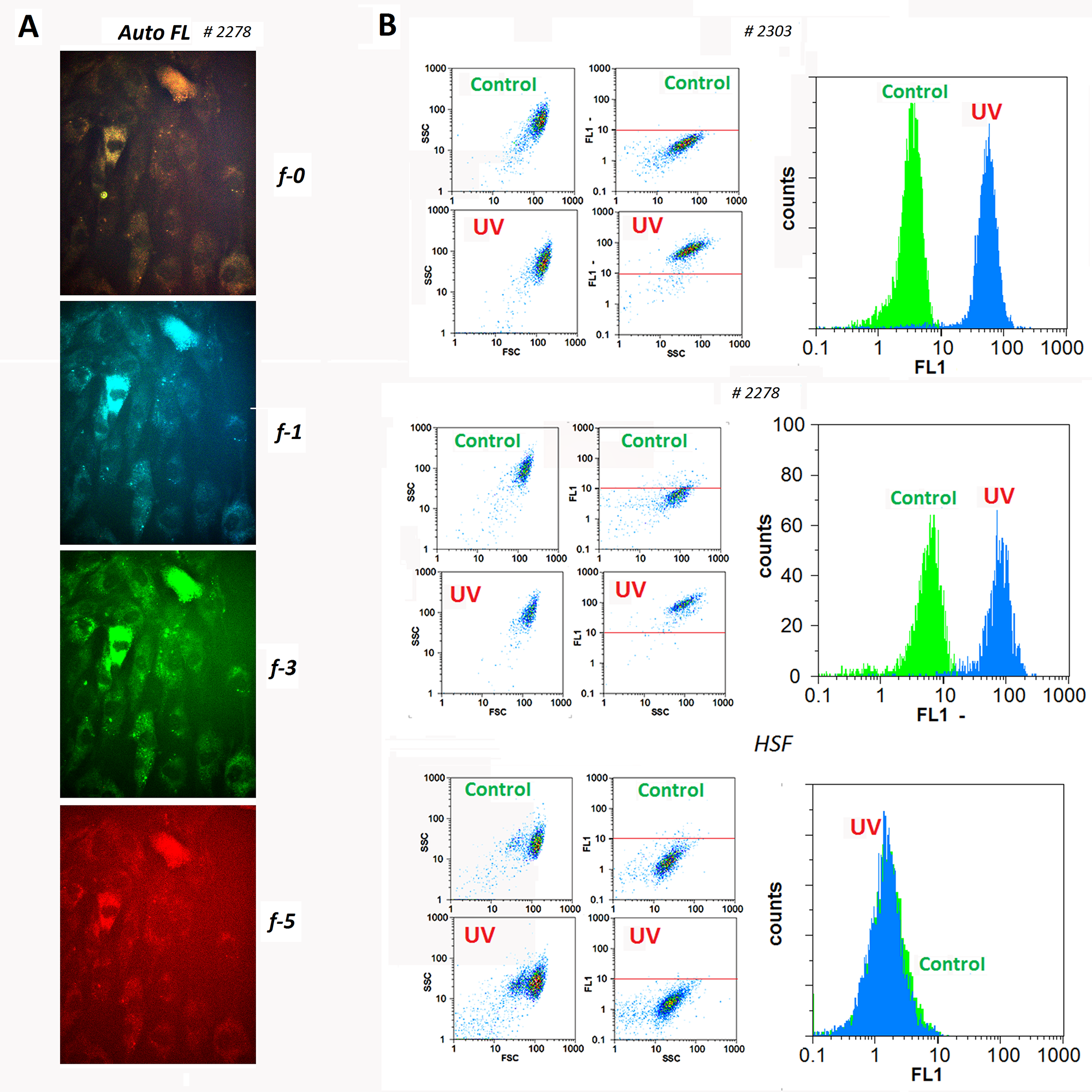

Supplement: Supplementary Figure 1 — (A) Autofluorescence of the irradiated MSCs (10 cGy) #2278. Photographs were taken with various fluorescent filters. (B) FACS. UVa irradiation effect on the MSCs and HSFs auto- fluorescence. [file Image_1.TIF]

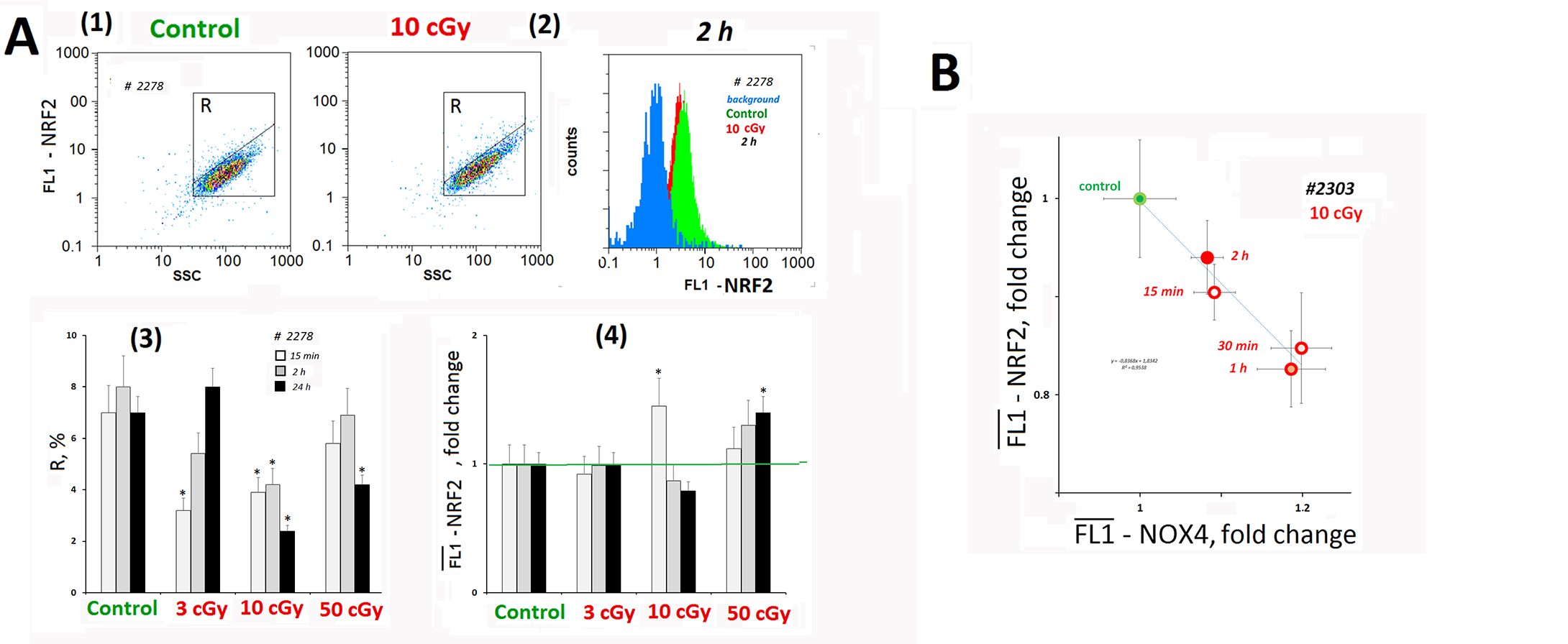

Supplement: Supplementary Figure 2 — (A) FACS. (1) Plots: FL1-NRF2 versus SSC. R: gated area, cells with an increased NRF2 expression. (2) The distribution of the MSCs (10 cGy) with varying NRF2 signal. (3) The content of the cells with an increased NRF2 expression. (4) Median signals for FL1 – NRF2. Average values and SD are given for three experiments. ∗p < 0.001 (U test). (B) Fluorescence microscopy. The dependence of FL1- NRF2 on FL1 – NOX4 changes in #2303 MSC (10 cGy). [file Image_2.TIF]
